# Nonalcoholic Fatty Liver Disease/Non-Alcoholic Steatohepatitis in Childhood: Endocrine-Metabolic “Mal-Programming”

**DOI:** 10.5812/hepatmon.17641

**Published:** 2014-05-01

**Authors:** Sara Manti, Claudio Romano, Valeria Chirico, Martina Filippelli, Caterina Cuppari, Italia Loddo, Carmelo Salpietro, Teresa Arrigo

**Affiliations:** 1 Department of Pediatric Sciences, Genetics and Pediatric Immunology Unit, University of Messina, Messina, Italy

**Keywords:** Non-Alcoholic Fatty Liver Disease, Mallory Bodies, Oxidative Stress

## Abstract

**Context:**

Nonalcoholic Fatty Liver Disease (NAFLD) is the major chronic liver disease in the pediatric population. NAFLD includes a broad spectrum of abnormalities (inflammation, fibrosis and cirrhosis), ranging from accumulation of fat (also known as steatosis) towards non-alcoholic steatohepatitis (NASH). The development of NAFLD in children is significantly increased.

**Evidence Acquisition:**

A literature search of electronic databases was undertaken for the major studies published from 1998 to today. The databases searched were: PubMed, EMBASE, Orphanet, Midline and Cochrane Library. We used the key words: "non-alcoholic fatty liver disease, children, non-alcoholic steatohepatitis and fatty liver".

**Results:**

NAFLD/NASH is probably promoted by “multiple parallel hits”: environmental and genetic factors, systemic immunological disorders (oxidative stress, persistent-low grade of inflammation) as well as obesity and metabolic alterations (insulin resistance and metabolic syndrome). However its exact cause still underdiagnosed and unknown.

**Conclusions:**

Pediatric NAFLD/NASH is emerging problem. Longitudinal follow-up studies, unfortunately still insufficient, are needed to better understand the natural history and outcome of NAFLD in children. This review focuses on the current knowledge regarding the epidemiology, pathogenesis, environmental, genetic and metabolic factors of disease. The review also highlights the importance of studying the underlying mechanisms of pediatric NAFLD and the need for complete and personalized approach in the management of NAFLD/NASH.

## 1. Context

Nonalcoholic Fatty Liver Disease (NAFLD) is the major chronic liver disease in the pediatric population (1). The incidence is 9.6% of children overall and in up to 38% of obese children (1). NAFLD includes a broad spectrum of abnormalities (inflammation, fibrosis and cirrhosis), ranging from accumulation of fat (steatosis) to non-alcoholic steatohepatitis (NASH) (2). Signs characterizing NASH are presence of inflammation, ballooning degeneration, Mallory bodies, necro-inflammation, and pericellular fibrosis (2).

The development of NAFLD in children requires the coexistence of multiple factors, including: race/ethnicity, gender, pubertal transition, risk of obesity and/or visceral adiposity, insulin resistance, and metabolic syndrome (3).

The ethnic and/or racial inequality in NAFLD incidence is not well known. Probably, it may be related to differences in genetic and environmental factors body, insulin sensitivity and adipocytokine profile. There is a minor prevalence of NAFLD in African-American population; although it is reported a major incidence of risk factors (such as obesity and insulin resistance) for fatty liver. Otherwise, there is a clear predominance of fatty liver disease among Hispanic youth (2). 

Although it is unclear whether sex is a risk factor for NASH in children, several population-based studies suggest that gender more common is male than female, with a female to male ratio of 1:2 (3). These differences could be related to the levels of circulating sex hormones (estrogen-testosterone ratio), hepatic expression of sex hormone receptors, and pattern of growth hormone secretion. In fact, it has been shown that estrogens act as anti-apoptotic, anti-fibrogenic, and anti-inflammatory agents. Especially 4-hydroxy estrone decreases lipid peroxidation, cholesterol (oxysterols) oxidation products, and reactive oxygen species (ROS) (4).

Although, there are case reports of fatty liver disease in children two to three years old; some studies suggest that the most frequent period of onset is pubertal transition, trough sex hormones levels, insulin resistance, higher serum triglycerides (TG) and lower serum high density lipoproteins levels (HDL) (4).

Abdominal obesity, strongly associated with visceral adiposity and insulin resistance are involved into steatosis and NASH in children. As an adult, hypertriglyceridemia, hypertension and low level of HDL-cholesterol have been also noted in children affected by NASH (5). 

## 2. Evidence Acquisition

A literature search of electronic databases was undertaken for the major studies published from 1998 to today. The databases searched were: PubMed, EMBASE, Orphanet, Midline and Cochrane Library. We used the key words: "non-alcoholic fatty liver disease, children, non-alcoholic steatohepatitis, and fatty liver".

## 3. Results

### 3.1. Histopathology

In children, NASH can occur in two histological subtypes: type one, includes ballooning degeneration, macrovesicular fat liver, polymorphonuclear leukocyte infiltration and perisinusoidal fibrosis; otherwise type two, only described in the pediatric patients, is characterized by fat liver with portal fibrosis and/or inflammation and periportal mononuclear leukocyte infiltration. Ballooning degeneration and perisinusoidal fibrosis are absents (6). It is not known the mechanism favoring to two hystologic phenotypes. Types 2 is predominant among younger patiens, males, subjects with severe obesity, and Asian- American- Hispanic children (6).

It has been proposed histologic scoring system. NASH-Clinical Research Network (NASH-CRN) suggested a scoring system composed of 14 histologic features; of which there are four semiquantitaitve (steatosis, lobular inflammation, hepatocellular ballooning, and fibrosis) and nine features semiquantitative (microvesicularsteatosis, acidophil bodies, microgranulomas, lipogranulomas, portal inflammation, pigmented macrophages, megamitochondria, Mallory hyaline, and glycogenated nuclei). NASH-CRN also includes a NAFLD Activity Score (NAS with range 0-8): less than 3 is considered “non-NASH”; result between 3- 4 is borderline; a range of 5 or more is evalueted “NASH”. However, NAS does not correlate well with prognosis (7).

Other score knwon as “the Brunt” based on the semiquantitative assessment. It recognizes three grades of activity: mild (also known as grade 1); moderate (also known as grade two); severe (also known as grade three) (8).

However, pediatric patients need to a ‘specific score’ for NAFLD. It was called “Pediatric NAFLD Histological Score” (PNHS). It includes: steatosis and lobular inflammation (from zero to three), ballooning and portal inflammation (from zero to two) (9).

The recognition of histological subtypes is important for the knowledge of disease pathogenesis and progression.

### 3.2. Pathogenesis

It is hypothesized that pathogenetic mechanisms is due to numerous factors. On 1998, Day et al. proposed a “two-hit” hypothesis (10). The first step is caused by insulin resistance, promoting steatosis, and an increased lipid peroxidation (3). Additional hits are oxidative stress (from antioxidants and glutathione depletion, mitochondrial dysfunction, hormonal imbalance, hypoxia (11)), lipotoxicity, inflammatory mediators as adipocytokines, and gut microbiota-derived lipopolysaccharide (LPS) leading to hepatocyte injury, inflammation, apoptosis and fibrosis. Therefore, it has been evalueted the possibility of multiple parallel hits (12).

### 3.3. Steatosis

Hepatic steatosis arises from an imbalance between input and output of triglycerides (TG). TGs result from three fatty acids (FA) molecules and one glycerol. FA derive from de novo synthesis, adipose tissue, and diet in the form of chylomicrons. Generally, 80% of the TG in chylomicrons is hydrolyzed, troughlipoprotein lipase (LPL), to free fatty acids (FFAs); 20% is only transfered to the liver, mostly bound to albumin (13). In liver FFAs can be oxidized to produce energy and ketone bodies in mitochondria, can reform TG, can be arranged in very-low-density lipoproteins (VLDL).

In addition, carbohydrate feeding also increases de novo synthesis of FFA from acetyl–coenzyme A (CoA). While insulin and glucose promote lipogenesis by transcription of peroxisome proliferator-activated receptor c (PPARc) and factor sterol regulatory element–binding protein–1c (SREBP-1c) (14), and transcription factor carbohydrate responsive element-binding protein (ChREBP), respectively. This latter favours expression of liver-type pyruvate kinase, providing more substrate for FFA and TG synthesis. Therefore unlike insulin-sensitive and normal weight people, patients affected by obesity and insulin-resistance release higher FA and glycerol percentages, promote hepatic accumulation of free fatty acids (FFA) and their esterification to form TG, decreased apolipoprotein B-100 synthesis and stimulate lipogenesis (15).

### 3.4. Oxidative Stress

Excessive lipid accumulation in liver is frequently accompanied by oxidative stress and endoplasmic reticulum (ER) responses. ER allows synthesis and release ofmembrane proteins. For this function are needed high concentrations of intra-ER calcium. Accumulation of FFAs, unesterified cholesterol, diacylglyceride and phospholipids induce a decrease of intra-ER calcium and an increase of“ER stress”,promoting apoptosis and hepatic stellate cellor Kupffer cell recruitment (12). High serum FFAs levels activate ketogenesis, mitochondrial, peroxisomal and microsomal FA oxidation. These are the main sources of reactive oxygen species (ROS), wich in turn contribute to apoptosis and to nuclear - mitochondrial DNA damage in NASH (12). 

Under physiological conditions, oxidative reactions convert oxidized (NAD+ and FAD) into reduced (NADH and FADH2) cofactors and increases electrons flow to chain. Otherwise, continuous oxidative stress creates a decreased outflow from respiratory chain, it in turns promotes higher serum ROS levels and lower anti-oxidant activity (12). Moreover, ROS, through polyunsaturated fatty acids (PUFA), promote release of 4-hydroxy-2-nonenal (4-HNE) and/or malondialdehyde (MDA) (12), diffusing into other sites and favouring oxidative stress. Thus, ER stress and mitochondria comprise (it is also impaired PPAR-γ and -coactivator-1α (PGC-1α) can further increase oxidative stress (16). 

### 3.5. Cytokines and Inflammation

FFA and ROS also promote production of inflammatory cytokines. Cai et al. demonstrated that hepatic steatosis leads to increased nuclear factor-κB (NF-κB: a transcription factor activated upstream by inhibitory κB (IKKβ) signalling in the liver). NF-κB induces the production of local and systemic inflammatory mediators (such as TGF-b, Fas ligand, TNF-a, leptin, adiponectin, IL-6, IL-1b, IL-8) (Table 1) involved in different lesions of NASH such as activation of Kupffer cells and macrophages, apoptosis, and inflammation. There is evidence that NF-κB can lead directly to insulin resistance (17). In addition, higher serum TNF-a levels, from adipocytes, promote mitochondrial permeability and b-oxidation, release cytochrome c, and increase electrons flow to respiratory chain. These events can futher conduct to creating ROS and promotingmitochondrial damage (18).

**Table 1.** Molecular Factors Promote Nonalcoholic Fatty Liver Disease/ Non-Alcoholic Steato-Hepatitis ^a^

Molecular Factors Promote NAFLD/NASH	Causes Induction	Effects	References
**NF-κB**	Hepatic Steatosis IKKβ	TNF-a, Fas ligand, TGF-b, IL-8, leptin, adiponectin, IL-6, IL-1b	Cai et al. (17)
		Activation of Kupffer cells and macrophages	
		Apoptosis/inflammation.	
		IR	
**TNF-a (and T-1031C/C-856A)**	Fat-engorged adipocytes Kupffer cells	Hepatic mitochondrial permeability	Cai et al. (17) Takaki et al. (12), Reinehr T et al. (19)
		B-oxidation	
		Release cytochrome c	
		Increase of electron delivery to the mitochondrial respiratory chain	
		Lipolysis and hepatic lipogenesis	
		Amplify hepatic synthesis of FA	
		Interferes on IRS-2 proteins, causing IR	
		Inhibits adiponectin expression	
		Promote secretion of leptin	
**IL-1**	Th-1, TLR-9	Promote secretion of leptin	Cai et al. (17) Roh et al. (20)
			
		IR	
		Hepatic fat accumulation	
		Acts on HSC to induce liver fibrosis	
**IL-2**	Th-1a	IR	Seth et al. (21)
**IL-6 (and 174G/C)**	NF-κBa	Lipolysis	Carulli et al. (22), Reinehr T et al. (19)
		Amplify hepatic synthesis of FA	
		MetS	
**IL-8**	NF-κBa	Activation of Kupffer cells and macrophages	Cai et al. (17)
		Apoptosis	
		Inflammation	
**TGF-b5**	NF-κB2	Activation of Kupffer cells and macrophages	Cai et al. (17)
		Apoptosis	
		Inflammation	
**IFN-a**	Th-1	IR	Seth et al. (21) Pacifico et al. (23)
		Proatherogenic effects	
**HMGB1**	Hepatocytes	Inflammation	Salpietro C et al. (24), Lin YC et al. (25), Arrigo et al. (26)
		Increased TLR-4	
		Fibrosis	
**TLR-2**	Expressed on monocytes, myeloid dendritic cells or mast cells	Inflammation.	Roh et al. (20) Salpietro C et al. (27)
		Fibrosis	
**TLR-4**	HMGB1	Inflammation	Roh et al. (20)
		Fibrosis	
**TLR-9**	Expressed on Kupffer cells	Promote release IL-1b	Takaki et al. (12)

^a^ Abbreviations: FA, fatty acids; HMGB1, high mobility group protein B1; HSC, hepatic stellate cell; IKK-B, inhibitory- kappa B; IL, interleukin; INF-a, interferon-alpha; IR, insuline resistance; IRS-2, insulin receptor substrate-2; MetS, metabolic syndrome; NAFLD, nonalcoholic fatty liver disease; NASH, non-alcoholic steato-hepatitis; NK-κB, nuclear factor- kappa B; TGF-b, transforming growth factor- beta; Th-1, T-helper-1; TLR, toll like receptor; TNF-a, Tumor necrosis factor-alpha.

### 3.6. NASH and Gut Micriobiota

Gut microbiota play a critical role in NAFLD and NASH (12). Recently, it has been demonstrated that gut microbiota of obese patients presents alterations in the microbial composition. It, through disrupted intercellular tight junctions and/or other proinflammatory bacterial products, can favour intestinal inflammation and permeability (28). Precisely, the link between gut microbiota, liver inflammation and immune system involves TLRs, important mediators between environment and immunological response (27) and endogenous substances such as short-chain fatty acids and high mobility group protein B1 [HMGB1] (20) Table 1. This latter plays an important role in the inflammatory process associated in several diseases (24) such as obesity (26).

TLRs involved in the pathogenesis of NASH are TLR2 (for lipoproteins and glycolipids in bacteria adhering to myeloid dendritic cells mast cells or monocytes), TLR4 (for palmitic- stearic and lauric- acid, and LPS of B cells myeloid dendritic cells, mast cells, monocytes and intestinal epithelium) (20), and TLR9 (for unmethylatedCpG DNA- bacterial particles) (12) Table 1.

TLR2 mediates liver inflammation and fibrosis (20). 

In steatosis hepatic, HMGB-1, releasing from hepatocytes in response to FFAs, is promoting an increased TLR4 protein levels and release of hepatic pro-inflammatory and pro-fibrogenic cytokines. This is achieved by inducing reactive ROS-dependent activation of X-box binding protein-1 (XBP-1) (29).

Studies conducted on choline-deficient amino acid-defined (CDAA)- murine NASH model, suggest that activation of TLR9 on Kupffer cells promotes release of IL-1b, which in turn stimulates hepatic fat accumulation. IL-1b also activates HSCs to induce liver fibrosis (20).

### 3.7. NASH and Genetic

Genetic predisposition and environmental factors contribute to an individual's susceptibility to develop hepatosteatosis, however their specific contributes in NASH are not clearly understood (30). Several observational studies on NASH suggest a strongly genetic contribution. Numerous candidate genes have been selected largely based upon the "two-hit hypothesis" of the pathogenesis of NAFLD, although other hypothesis-independent approaches can also be informative in gene selection (31). Kahle et al. focused on genotype-dependent and -independent adaptations early in the pathogenesis of hepatosteatosis by characterizing C3HeB/FeJ, C57BL/6NTac, C57BL/6J and 129P2/OlaHsd in mice (30). Several studies showed ethnic-genetic factors as well as a link between hepatic steatosis and hepatic inflammation, and insulin resistance (IR) or type 2 diabetes mellitus (T2D) in combinationwith obesity (31). Recently, authors get their attention on PNPLA3 rs738409 C > G (I148 M), SOD2 rs4880 C > T, KLF6 rs3750861 G > A, and LPIN1 rs13412852 C > T polymorphisms. These were detected in obese children with increased liver enzymes (32). 

The function of PNPLA3, which encodes the I148M protein variant in the catalytic domain, is not well known. However, Matteoni and colleagues demonstrated that the PNPLA3 gene is closely related with disease progression (33). In fact, pediatric patients with high dietary omega6/omega3 PUFA intake (34) and with single nucleotide polymorphism (SNP) rs738409 G/G genotype, could evolve from fat liver, hepatocellular ballooning, lobular inflammation, and perivenular fibrosis to NASH. Furthermore, PNPLA3 could act as a downstream gene of sterol-regulated binding protein 1c (SREBP-1c) to promote lipid accumulation. New results provide an evidence that PNPLA3 is alsoassociated with lower HDL cholesterol (33). 

Unlike, variants near NCAN (which encodes for an adhesion molecule), PPP1R3B (which encodes for a protein that regulates glycogen breakdown) and GCKR -SNP rs1260326- (which, through inhibition of glucokinase, regulates glucose storage/disposal and provides substrates for de novo lipogenesis, are associated with distinct changes in serum and liver lipids as well as glycemic traits (35). Other SNP are useful for the search of genetic factors responsible for diseases. MTP -493 G/T polymorphism may impact NASH by modulating postprandial lipemia and lipoprotein metabolism; homozygous GG carriers have a more atherogenic postprandial lipid profile, independently of adipokines and IR (36).

SNPs may influence the resistin gene expression. It is regulated by CCAAT/enhancer binding protein (C/EBP) α and peroxisome proliferator-activated receptor (PPAR)-γ in the phosphatidyl inositol 3-kinase (PI-3 K) and mitogen-activated protein kinase (MAPK) pathways, inhibited the role of insulin in glucose uptake and impaired glucose tolerance (37). The resistin + 299AA genotype may be associated with increases in the risk of the NAFLD development in T2DM patients (38). Resistin may also upregulate the suppressor of cytokine signaling (SOCS)-3 gene expression and repress the insulin receptor substrate (IRS)-2 gene expression, leading to impaired glucose tolerance in cells (38). SNP variants in terpenoid synthesis, cholesterol biosynthesis and biosynthesis of steroids were associated with lobular inflammation and cytologic ballooning while those in terpenoid synthesis were also associated with fibrosis and cirrhosis (38).

It also has been hypothesized that increased hepatic expression of CYP2E1 (variant allele CYP2E15), carries out omega hydroxylation of fatty acids, leads to increased levels of toxic lipid peroxides and its possible increased expression in NASH (39). 

Other genetic variation in lipid metabolism involves microsomal triglyceride transfer protein (MTP), an enzyme that regulates synthesis, storage, and export of hepatic triglyceride content. A common genetic variation of the MTP gene is –493 G/T polymorphism. It lead to lower export of TG from hepatocytes, and higher intracellular accumulation (40). Functional polymorphisms in phosphatidylethanolamine N-methyltransferase (PEMT), IL-1b, and manganese superoxide dismutase (MnSOD) have also been reported in Japan (41). 

It has been intensively investigated functional genetic polymorphisms of gene Kruppel-like factor 6 (KLF6) (42), IL-6 (174G/C) (22) and TNF-a (T-1031C and C-856A in the promoter region). It were more frequent in patients with NASH, mediating progression of the disease. 

The functional polymorphisms G45T and G276T in the adiponectin gene have been reported to be associated with diabetes. Regarding Japanese subjects with NASH, it has been noted that the G/G homo-allele at the 45th base of the exon of adiponectin was more frequent in NASH with advanced fibrosis and insulin resistance (43). 

Several study was also conducted on hepatoprotective gene component. Rossi et al. showed the role of Cannabinoid Receptor type 2 (CB2) in a large cohort of obese children. They have found that the CB2 Q63R variant correleted with severity of NASH, suggesting that CB2 Q63R variant has a critical role in modulating hepatic inflammation state, and the liver damage (43).

PPARα and PPARγ are members of a family of nuclear receptors involved in the metabolism of lipids and carbohydrates, adipogenesis and sensitivity to insulin. Domenici et al. documented that Pro12Ala SNP may result in protection against liver injury and that Leu162Val SNP may be involved in the progression of NAFLD (16).

The APOC3T-455C and C-482T promoter region polymorphisms have also hepatoprotective properties (44). Lin et al. hypothesized that variant UGT1A1 genotypes reduce the risk for NAFLD development. Variants of this gene contribute to increased bilirubin levels, acting as an antioxidant factor (25). In conclusion, these recent data may be useful to predict long-term outcomes of the disease and guide clinical management (14) especially for children with family members affected by NAFLD. Further studies are needed to investigate a possible role of genetic component in disease progression.

### 3.8. NASH and Obesity

Childhood obesity and obesity-related conditions are significantly increased. Obesity is a factor point to the multifactorial nature of NAFLD/NASH, however, only a subset of obese children develops NAFLD. In North America, Europe and Asia, the prevalence of NAFLD is between 10 and 77% (45). In fact, higher Body Max Index (BMI), strongly and independently of other risk factors, increases the risk of liver fibrosis in young population (46).Similar to adult data, abdominal obesity with visceral obesity might be predicting factor for accumulation fatty liver. Traditionally, in the context of obesity, it was considered that lipid accumulation in the liver is coming from an elevated plasma NEFA pool promoted by increased lipolysis and from activity of hormonesensitive lipase (47). Adipose tissue is also recognized as an immune organ that secretes numerous immunomodulatory factors and seems to be a significant source of inflammatory signals. The consequent release of these molecules (adipokines, Tumor Necrosis Factor alpha (TNF-a) adiponectin and resistin), is one of the earliest protagonists involved in the development of IR and low-grade inflammation (4). 

Leptin, produced in adipocytes, regulates satiety and metabolism at the hypothalamic and peripheral level (48). Serum leptin levels are also influenced by sex hormones: testosterone inhibits them, otherwise ovarian sex steroids increase them. SimilarlyIL-1 and TNF-a promote the release of leptin (12). Leptin may contribute toenhance hepatic steatosis by changing actions of insulin on tissues and its receptors, and it may influence the development of NASH through the regulation of inflammatory responses (48). Seth et al. demonstrated that higher levels of oxidative stress-induced leptin mediated CD8+CD57+ T cells play an important role in the development of NASH. In fact, there was a significant increase in the levels of Th1 cell cytokines IL-2, IL-1b, and IFN-γ highly correlated with to IR as well as to NASH, independently of anthropometric feature (21) (Table 1).

Moreover, Th1- secreted IFN-y has also proatherogenic effects. It is an immune-activating cytokine that promote an inflammatory response, such as activation of macrophages, delayed-type hypersensitivity, and granulomatous lesions. Therefore, it also might provide an important mechanism for liver damage (23). Most obese humans, with steatosis and/or NASH, present elevated serum leptin levels, because of leptin resistance (48). Adiponectin is an adipose-specific hormone having anti-inflammatory, insulin-sensitizing, and antiatheriosclerotic effects (49). As in adults, observational pediatric data revealed that serum adiponectin levels and adiposity, IR and hepatic fat are inversely correlated (50). In fact, it is showed that adiponectin protects from TG accumulation in hepatocytes. It promotes b-oxidation of FFAs and decreas de novoTG synthesis (51). Thus, hypoadiponectinemia plays a crucial role in disease progression and in development of metabolic syndromes. Probably, adiponectin can be a biomarker for insulin sensitivity. Resistin also antagonizes insulin action, causing glucose intolerance (50), whereas elevated serum resistin levels are associated with IR (52). This latter is also increased by TNF-a and IL-6. In fact, they indirectly mediate lipolysis and amplify hepatic synthesis of FA (19). TNF-a, produced by adipocytes in visceral fat and Kupffer cells in the liver (12), interferes on IRS-2 proteins, causing IR. Besides, TNF-a inhibits adiponectin expression, causes high serum FFAs levels through stimulation of lipolysis and hepatic lipogenesis, and it may lead to development of NAFLD (2, 53).

### 3.9. NASH and Insuline Resistence (IR)

The common link between obesity and NAFLD/NASH is insulin resistance (IR), this latter is promoting peripheral lipolysis and de novo lipogenesisfavouring an increased flux of FFAs into the liver (54). It has been noted a higher incidence of IR in young obese with NAFLD (55) and that those with NASH had a higher likelihood of having abnormal mitochondrial morphology suggesting a increased oxidative stress (56). The molecular mechanism promoting to IR is complex and it has not been well elucidated (10). Hepatic fat is one proposed mechanism of IR, although it is not also known if IR is a cause or consequence of lipid accumulation (54).

In healthy state, insulin stimulation of IRS-2 in hepatic cells leads to activation of intracellular PI3K which in turn activates glucose transporter (GLUT) allowing glucose entry. Otherwise, in IR- conditions, the adipose tissue does not respond to the antilipolytic of insulin. Therefore, the flux of FFAs into the liver is increased (57). Therefore, circulating FFAs, derived from peripheral and visceral adipose tissue, promote increase in intracellular metabolites (diacylglycerol) which in turn leads to decreased phosphorylation of IRS-2, of PI3K and dysfunctional cellular glucose production (58). Hyperglycemia further promote lipid accumulation in hepatocytes by stimulating lipogenesis.Moreover, IR, through depletion of hepatic n-3 polyunstaurated fatty acids (PUFAs), favours imbalance lipidic metabolism. In fact, n-3 PUFAs plays a key role in regulating the metabolic switch from anabolism (lipogenesis) to catabolism (Fatty Acid Oxidation) by activating PPARα, a positive regulator of FAO (59). 

IR is also involved in the development of fibrosis by increasing fatty acid β-oxidation and oxidative stress (46). Several cross sectional studies have found strong and positive association between IR and severity disease (60), confirming a criticalpathophysiological role of IR in the development of NAFLD. Ko et al. reported that 96% of their pediatric patients with NAFLD demonstrated IR (HOMA-IR > 2) (61). Chan et al. showed a positive correlation between IR and male obese children affected by NASH (62). Adipo-IR (FFA × INS) index quantifies Adipose tissue-IR ratio (63). It reflects the inability of insulin to decrease peripheral lipolysis. Patients affected by NAFLD, even if not obese, showed increased FFA concentrations and Adipo-IR (64). Probably, IR can be a biomarker for hepatic liver damage (65).

### 3.10. NASH and Metabolic Syndrome (MS)

On 1998, World Health Organization coined the term “metabolic syndrome” (66). The main features of metabolic syndrome (MetS) are: central obesity (waist circumference greater than 102 cm for males, and greater thab 88 cm for females), systolic and/or diastolic blood pressure ( > 95th percentile), low serum HDL-cholesterol levels (less than 5th percentile), high serum TG levels (greater than 95th percentile), and impaired fasting glucose (greater than 100mg/dl) (67). In children, it has also been demonstrated a relationship between MetS and NAFLD (18% in normal-weight to 67% in obese subjects) (68). The Korean National and Nutrition Examination Survey found that young people 10-19 years old, with three or more risk factors for MetS, have an higher serum alanine aminotransferase levels, indicating steatosis (69).

The higher indicence of MetS among children affected by NASH can be explained by the fact that obese/overweight patients were presenting with at least one hepatic abnormality (clinical hepatomegaly and/or raised ALT) (70). It has also been hypothesized that NAFLD might be thehepatic feature of MetS (71). The pathogenesis of MetS and NAFLD are incompletely understood. The overlap of potential mechanisms provides insights into their pathogenesis. NAFLD requires an hepatic accumulation of FFA e TG. IR suppresses glycogenesis, promotes gluconeogenesis and triglyceride synthesis. Progression to NASH involves oxidative stress and the release of inflammatory cytokines. These inhibits fatty acid uptake, stimulates fatty acid oxidation and lipid export and enhances insulin sensitivity (72).

According to Reineh et al. Fetuin-A protein, produced by liver, could be a link between insulin resistance, obesity, and MetS in NAFLD. Hepatic TG accumulation causes acquired insulin-signaling defects (probably via Fetuin-A) and subsequent IR, glucose intolerance and Type 2 diabetes mellitus. To confirm this, obese children with NAFLD have significantly elevated fetuin-A concentrations compared with obese children without NAFLD and control group (73). It must also consider that adipocytes, regulated by insulin, represent a relevant source of numerous peripheral and neuroendocrine peptides such as polactin (PRL). PRL plays a pivotal role in metabolic balance, acting on adipogenesis, lipolysis and release of adipokines as well as IL-6 and adiponectin (74), involved in the pathogenesis of MetS and NASH.

## 4. Conclusions

Pediatric NAFLD/NASH is emerging problem. However, its exact cause, prevalence and progression still underdiagnosed and unknown. Further studies will bring new insight into this complex disorder for the development of novel diagnostic and therapeutic strategies that might enable a personalized approach in the management of NAFLD/NASH.
